# Differentiated Thyroid Cancer, From Active Surveillance to Advanced Therapy: Toward a Personalized Medicine

**DOI:** 10.3389/fendo.2019.00884

**Published:** 2020-01-08

**Authors:** Antonio Matrone, Maria Cristina Campopiano, Alice Nervo, Giulia Sapuppo, Martina Tavarelli, Simone De Leo

**Affiliations:** ^1^Endocrinology Unit 1, Department of Clinical and Experimental Medicine, University of Pisa, Pisa, Italy; ^2^Oncological Endocrinology Unit, Department of Medical Sciences, School of Medicine, Cittá della Salute e della Scienza Hospital, University of Turin, Turin, Italy; ^3^Division of Endocrinology, Department of Clinical and Experimental Medicine, Garibaldi-Nesima Medical Center, University of Catania, Catania, Italy; ^4^Division of Endocrine and Metabolic Diseases, IRCCS, Istituto Auxologico Italiano, Milan, Italy

**Keywords:** differentiated thyroid cancer, active surveillance, radioiodine (^131^I) treatment, tirosine kinase inhibitors, dynamic risk stratification

## Abstract

Differentiated thyroid cancer (DTC) is the most frequent endocrine malignancy and represents the most rapidly increasing cancer diagnosis worldwide. In the last 20 years, this increase has been mostly due to a higher detection of small papillary thyroid cancers, with doubtful effects on patients' outcome. In fact, despite this growth, cancer-related death remained stable over the years. The growing detection of microcarcinomas associated to the indolent behavior of these cancers led to the development of strategies of active surveillance in selected centers of different countries. Moreover, toward a more personalized approach in the management of DTC patients, surgical treatments became more conservative, favoring less extensive options in patients at low risk of recurrence. The rise in lobectomy in low-risk cases and the need to avoid further therapies, with controversial impact on recurrences and cancer-related death in selected intermediate risk cases, led to reconsider the use of radioiodine treatment, too. Since clinicians aim to treat different patients with different modalities, the cornerstone of DTC follow-up (i.e., thyroglobulin, thyroglobulin autoantibodies, and neck ultrasound) should be interpreted consistently with this change of paradigm. The introduction of novel molecular target therapies (i.e., tyrosine kinase inhibitors), as well as a better understanding of the mechanisms of immune checkpoint inhibitor therapies, is radically changing the management of patients with advanced DTC, in whom no treatment option was available. The aim of this review is to analyze the most recent developments of the management of DTC, focusing on several key issues: active surveillance strategies, initial treatment, dynamic risk re-stratification, and therapeutic options in advanced DTC.

## Introduction

The recent advances in knowledge about differentiated thyroid cancer (DTC) showed the need of a personalized management approach. Despite the increased diagnosis of DTC, in particular small papillary thyroid cancers, cancer-related death remained stable over time. The clinical challenge is moving toward the identification of patients with indolent tumors, who can be treated and followed with a more conservative approach, as opposed to those in which aggressive therapy and intensive follow-up should be recommended. This paper discusses the key points of DTC management, from active surveillance to advanced therapy.

## Active Surveillance

Active surveillance has been proposed as an alternative to immediate surgery to avoid overtreatment in unifocal intrathyroidal papillary microcarcinoma (mPTC), without metastatic lymph nodes or aggressive cytological features (1).

Many retrospective papers showed the excellent outcome of mPTC over time (2, 3) likely related to the indolent behavior of mPTC, rather than the efficacy of treatments. For the first time at Kuma Hospital in Japan, in 1993, a conservative approach for selected mPTC patients was hypothesized, and then, active surveillance was introduced in clinical practice (4).

To date, in the Japanese series, active surveillance has the same outcome of immediate surgery. After 10-year observation, only 8 and 3.8% of 1, 235 mPTC under active surveillance showed nodule enlargement or appearance of lymph node metastases, respectively (5). None of the patients who underwent surgery after disease progression showed higher rates of recurrence or mortality compared to patients who performed immediate surgery (5).

Neither clinical nor pathological features, like sex, familial history of thyroid cancer, and multifocality, were related to mPTC progression (6). Conversely, the estimated lifetime probability of mPTC progression was related to the age at diagnosis, being 60.3% for younger (25 years) and 3.5% for older patients (75 years) (7). Therefore, older mPTC patients represent the best candidates for active surveillance. However, ~40% of younger patients could be safely followed up without requiring surgical treatments during lifetimes, corroborating the feasibility of active surveillance.

Active surveillance is a likely safe and feasible strategy also in pregnancy: only 8% of pregnant women showed mPTC progression, and the following postpartum thyroidectomy was completely curative (8).

Based on the Japanese experience, active surveillance was introduced in mPTC management also in other countries, confirming the safety of this approach (9–11). It was observed that mPTC changes in volume during time, rather than in maximum diameter, represent a more sensitive tool to select those patients who could benefit from a more careful monitoring or surgical treatment. Currently, this conservative approach in clinical practice is mainly limited by the burden of cancer diagnosis, its impact on patients' everyday life, and the wide skepticism shown also by general practitioners, different specialists, and institutions (11–13). The assessment of the quality of life and the long-term results of active surveillance, also outside Japan, might improve the patients' acceptability and their compliance in future.

A risk stratification of mPTC patients according to a clinical framework (14, 15) has been proposed: clinical features (nodules with well-defined margins without evidence of extrathyroidal extension and cN0, cM0) and sociocultural and psychological evaluation (willing to accept this approach, aware of possible surgical treatment in the future, compliant to follow-up) are critical to identify the ideal candidate for active surveillance.

Several issues remain open, such as the ideal frequency of follow-up evaluations, currently recommended every 6 months for the first 2 years and yearly afterwards, the optimal timing for surgery in progressive cases, the availability of more precise radiologic tools to improve the detection of minimal extrathyroidal extension, and the identification of molecular biomarkers. To date, the role of genetic mutations (e.g., BRAF, RAS, TERT, etc.) and/or rearrangements (such as RET/PTC) are not completely understood in mPTC during active surveillance. These biomarkers could be able to distinguish the more aggressive forms of mPTC.

## Initial Treatment

### Surgery

Accurate preoperative staging is essential to evaluate the primary tumor and the presence of lymph node metastasis to guide the extent of surgical treatment. Clinical and ultrasonographic evaluation of the neck (nUS) is the cornerstone of the initial assessment. Cross-sectional imaging (i.e., CT scan with IV contrast) might be useful in selected, locally advanced cases.

Many changes in the extent of surgery for DTC have been recently advocated. In the past (16), total thyroidectomy (TTx) was recommended for all DTC >1 cm, regardless of other pathological features, based on several studies that showed lower recurrence rates in patients treated by TTx compared to lobectomy (17–19). Currently, lobectomy is recommended for low-risk and well-differentiated unifocal thyroid cancer ≤4 cm (1, 20). In this setting, the use of nUS before surgery is essential: any suspicion of extrathyroidal extension, multifocal disease, or lymph node metastasis should lead to TTx. Lobectomy can avoid postoperative hypoparathyroidism and the need of thyroid hormone replacement (21). Conversely, in patients who perform TTx, there is no need for completion thyroidectomy, thyroglobulin (Tg) can be used as marker of recurrence, and ^131^I treatment can be performed, if needed.

For all DTC >4 cm, or ≤4 cm with aggressive features, TTx is usually recommended (1, 20).

In the presence of clinically evident lymph node metastasis of the central or latero-cervical compartment, an oriented therapeutic lymph node dissection is recommended (1, 22–26) because it is associated with lower recurrence (27) and disease-specific mortality rates (28, 29). Prophylactic lymph node dissection of central compartment is still debated and, to date, not routinely recommended (1, 20).

## Postoperative Radioiodine Treatment With ^131^I

Radioiodine treatment with ^131^I (RAI) is not generally recommended after lobectomy (1, 20). After TTx, RAI could be used for three different purposes (30):

1. Remnant ablation: to eliminate any thyroid tissue/cells left over the surgery, making the measurement of serum Tg more specific for persistent/recurrent disease;
2. Adjuvant treatment: to eliminate any potential foci of thyroid cancer, which might be present after surgery and to destroy small-volume microscopic lymph node metastasis;
3. Treatment of known disease: to treat the tumor residual disease in the case of advanced stage, both at local and distant level.

Moreover, RAI treatment allows to perform a posttherapeutic whole-body scan, with or without single-photon emission CT–CT (SPECT-CT), to evaluate the presence of radio avid local or distant metastasis, which could change the initial staging and theoretically further treatments (31).

Initial treatment of DTC consisted of TTx plus RAI for all patients until few years ago; nowadays, patients treated with RAI should be selected on the basis of the initial risk stratification (low, intermediate, and high) (1, 20, 32). Furthermore, the decision making to perform RAI should be in agreement with postoperative evaluation, too (1, 33–35). To increase thyroid-stimulating hormone (TSH) levels (36), to rise ^131^I uptake in thyroid cells, RAI can be performed after thyroid hormone withdrawal (THW) or administration of recombinant human TSH (rhTSH).

In high-risk patients, routine postoperative RAI with high activities is required because it improves the specific cancer survival (37). Activities up to 150 mCi are generally recommended when RAI is used as remnant ablation or adjuvant therapy. In the presence of known distant metastasis, higher activities can be used (1, 20). Furthermore, major guidelines (1, 20, 26) suggest THW preparation, while more data are needed before recommending for or against rhTSH.

In low- and intermediate-risk cases, two randomized non-inferiority trials comparing low and high activities of radioiodine, each with either THW or rhTSH, demonstrated that the ablation rate was similar in the four groups (38, 39). In addition, recurrence rates are similar in patients prepared with THW or rhTSH, both in low- (40, 41) and intermediate-risk patients (42). However, the preparation with rhTSH significantly improves quality of life (43, 44) and reduces both whole-body irradiation (45) and hospitalization time (46, 47).

Selective use of RAI is advocated for patients with intermediate risk. Aggressive variants have a worse prognosis than classic variant, and RAI increased cancer-specific survival rate (48, 49). The role of microscopic extrathyroidal extension (mETE) in deciding to perform RAI or not is debated; some studies demonstrated a risk of death (50), while others did not (51). The presence of BRAF V600E mutation is associated with increased cancer-specific mortality (52) and a higher risk of recurrence (53) compared to wild-type tumors. In intermediate-risk BRAF-positive tumors, reasonably, the associated histological features have more relevance in deciding to perform RAI, compared to the presence of mutation alone. Therefore, RAI should be performed, with low or high activities (i.e., 30–150 mCi) and after rhTSH or THW, in selected intermediate-risk cases with advanced age, aggressive histology, and higher volume of lymph node metastasis (1, 20). In other clinical scenarios (i.e., mETE, small lymph node metastasis, and intrathyroidal PTC with BRAF mutation), postoperative evaluation should guide the decision (1, 20).

In low-risk cases, including those with positive BRAF mutation and small volume metastatic lymph node involvement, RAI is not recommended anymore because the cancer-specific mortality and the persistent/recurrent disease is negligible and therefore not improved by RAI (54–56). Accordingly, based on the postoperative evaluation and in selected cases, RAI should be performed with low activity (i.e., 30–50 mCi) and after rhTSH stimulation (1, 20).

## Dynamic Risk Restratification

### Follow-Up After Initial Treatment and Dynamic Risk Stratification (DRS)

In the last few years, DTC follow-up has changed. Differently from the past, dynamic risk stratification (DRS) is widely performed at both the first postoperative and the subsequent evaluations, and it takes into account information obtained during follow-up (1).

### After Lobectomy

DTC is often multifocal and bilateral and is inclined to spread to the loco-regional lymph nodes. After lobectomy, all patients should undergo basal serum Tg and TgAb measurement and periodical nUS. When patients are carefully selected to surgery, the recurrence rate is low, ranging from 1 to 7%, with no impact on overall survival (1, 57, 58). In addition, if properly treated, most of these patients remain free of disease also after recurrences.

A predefined threshold value of Tg, to recognize those patients with an incomplete response after initial treatment or with recurrence, is difficult to obtain because of the presence of the remaining lobe. For the same reason, TgAb trend loses its meaning during follow-up. However, it could be argued that a stable, non-stimulated value of Tg <30 ng/dl, in the absence of a suspicious nUS, could be predictor of an excellent response (59). On the contrary, a rising trend of Tg may predict a structural disease.

Similarly to TTx, in patients treated by lobectomy, response to treatment can be divided into four classes (20, 59):

1. Excellent response: stable basal serum Tg levels related to the presence of a contralateral thyroid lobe and negative neck US;
2. Biochemical incomplete response: basal serum Tg not related to the presence of a contralateral thyroid lobe, or increasing basal serum Tg levels without evidence of structural disease;
3. Structural incomplete response: evidence of structural disease;
4. Indeterminate response: non-specific findings on neck US and doubtful trends of Tg.

Accordingly, clinicians should tailor the intensity of treatment and follow-up.

#### TSH Targets During Short and Long-Term Follow-Up

In these patients, little evidence is available on the TSH level to be maintained during follow-up. In low-risk patients treated by lobectomy, TSH should be maintained between 0.5 and 2 mU/l, and levo-thyroxine is generally not recommended for all patients with a TSH value ≤2 mU/l (1, 20). In iodine-deficient areas, levo-thyroxine can be indicated according to patient age and comorbidities to reduce nodular hyperplasia of the remaining lobe (20).

### After TTx With or Without RAI

Postoperative evaluation should be performed after 4–12 months from diagnosis and includes Tg, using high-sensitive Tg assay, TgAb measurement, and nUS (33–35). Additional imaging evaluations could be useful in selected cases.

Patients are re-evaluated over time according to the DRS and are classified into four groups, regardless of RAI (1, 20, 32, 59):

1. Excellent response: non-stimulated Tg <0.2 ng/ml (both for TTx and TTx + RAI) and/or stimulated Tg <1 (TTx + RAI) or 2 ng/ml (TTx alone) plus undetectable TgAb and negative imaging;
2. Indeterminate response: non-stimulated Tg 0.2–1 ng/ml (TTx + RAI) or 0.2–5 ng/ml (TTx alone) and/or stimulated Tg 1–10 ng/ml (TTx + RAI) or 2–10 ng/ml (TTx alone) and TgAb levels stable or declining, in the absence of structural or functional disease or non-specific findings on imaging studies or faint uptake in thyroid bed on RAI scanning;
3. Biochemical incomplete response: non-stimulated Tg ≥1 ng/ml (TTx + RAI) or >5 ng/ml (TTx alone) and/or stimulated Tg ≥10 ng/ml or rising TgAb levels plus negative imaging;
4. Structural incomplete response: structural or functional evidence of disease regardless of Tg or TgAb levels.

The risk of recurrence depends on DRS status rather than initial risk category. The impact of DRS has likely more relevance for intermediate and high-risk patients with an excellent response during follow-up (decreased risk of recurrence up to 1–2%) (60–62).

#### TSH Targets During Short and Long-Term Follow-Up

The appropriate degree and duration of TSH suppression remains to be established. Currently, the international guidelines (1, 20) suggest a degree of TSH suppression according to the risk classification after initial treatment:

1. High risk: TSH ≤0.1 mU/l
2. Intermediate risk: TSH 0.1–0.5 mU/l
3. Low risk: TSH in the normal range (0.5–2 mU/l).

Despite an improved outcome in high-risk patients, currently, the use of TSH suppression is being reconsidered both in intermediate- and high-risk cases (63); no evidence of benefits has been documented in low-risk cases (55, 64). Moreover, in patients with associate comorbidities (at high risk of adverse effects by TSH suppression therapy), TSH values should be individualized, balancing risks, harms, and benefits (65).

TSH values should be titrated also after the restratification process during the follow-up:

1. Excellent response (clinically and biochemically free of disease): TSH 0.5–2 mU/l (in patients with high-risk disease at diagnosis, TSH 0.1–0.5 mU/l during the first 5 years)
2. Biochemical incomplete or indeterminate response: TSH 0.1–0.5 mU/l
3. Structural incomplete response: TSH ≤0.1 mU/l.

## Advanced Therapy

Despite patients with DTC usually having an excellent prognosis, a small subgroup could develop distant metastasis or become radioiodine refractory (RAI-R), with a significant impact on survival rates (66). There is no fully consensus on the definition of RAI-R disease (67). According to the American Thyroid Association guidelines (1), it refers to the absence of radioiodine uptake in all or some lesions, at the first posttherapeutic whole-body scan or after previous evidence of RAI-avid disease, or in the case of progression of disease despite RAI uptake. Furthermore, also the existence of a maximum cumulative RAI dose to be administered is still debated. These patients with a structural disease should be assessed by biochemical and imaging evaluation. Tg may give an estimate of tumor burden, and Tg doubling time <1 year is associated with a poor prognosis (68). However, imaging techniques (US, MRI, and CT scan) provide the most precise information on tumor burden to evaluate tumor growth and define tumor progression according to the response evaluation criteria in solid tumors (69–71). Positron emission tomography with 2-deoxy-2-fluorine-18-fluoro-d-glucose/CT (_18_FDG-PET/CT) may provide additional prognostic information, since _18_FDG-PET positive lesions usually have a more aggressive behavior (72, 73).

Differences among the main referral guidelines regarding the management of RAI-R DTC are reported in Table 1.

**Table 1:** Recommendations of the American Thyroid Association (ATA) Guidelines (2015), Italian Consensus (2018), and National Comprehensive Cancer Network (NCCN) (2019) about the management of radioiodine-refractory locally advanced/metastatic differentiated thyroid cancer (DTC) patients.

**Radioiodine-refractory locally advanced/metastatic DTC**			
	**Active surveillance**	**Locoregional treatments**	**Systemic treatments**
ATA 2015	Serial radiographic imaging (every 3–12 months) in patients with asymptomatic, stable, or minimally progressive disease, not likely to develop rapidly progressive, clinically significant complications. TSH-suppressive thyroid hormone therapy to be continued.	In case of symptoms and risk of local complications before systemic treatment (or during systemic therapy in case of progression of a single lesion): surgery, external beam radiotherapy (EBRT), percutaneous approach (i.e., radiofrequency, laser ablation, ethanol injection, cryoablation, cementoplasty) in selected cases.	Approved kinase inhibitor (KI; i.e., lenvatinib, sorafenib) in rapidly progressive, symptomatic, and/or imminently threatening disease not otherwise controlled using other approaches. Second-line KI therapy in case of progression or prohibitive adverse effects on first-line treatment (ideally within the context of clinical trials). Few data and disappointing results about conventional chemotherapy; to be considered after failure of KI therapy. Bisphosphonates (especially zoledronic acid every 3 months) or denosumab in case of diffuse and/or symptomatic bone metastases.
Italian Consensus 2018	Cross-sectional imaging at regular intervals (every 3–12 months) in case of stable disease without symptoms, with a slow progression during the follow-up and without lesions at risk of life. TSH-suppressive thyroid hormone therapy to be continued.	Strongly suggested in case of progression related to a single lesion treatable with a local and selective approach: surgery, EBRT, other local procedures (i.e., thermoablation, ethanol injection, chemoembolization).	Approved KI (i.e., sorafenib, lenvatinib) for rapidly progressive, significantly symptomatic, and/or with life threatening lesions not suitable for local therapies. In case of progressive disease during KI therapy, indication to another KI based on evidence of high probability of efficacy. Traditional chemotherapy only in case of failure or contraindication of KI.
NCCN 2019	In case of non-progressive and indolent disease, distant from critical structures. TSH-suppressive thyroid hormone therapy to be continued.	To be considered in case of progressive and/or symptomatic disease if feasible, depending of the site, and the number of tumoral foci: surgery, EBRT, other interventional procedures (i.e., ethanol ablation, cryoablation, radiofrequency, embolization) in selected patients.	Lenvatinib (preferred) or Sorafenib for progressive and/or symptomatic disease. Other commercially available KI to be considered if clinical trials not available or appropriate. Minimal efficacy of cytotoxic chemotherapy. Intravenous bisphosphonates or denosumab if bone metastases.

In general, in patients with oligometastatic, rapidly progressive, or symptomatic disease, a local treatment should be preferred. Surgery is the most widely used therapeutic procedure in these scenarios. Other techniques include thermal ablation (radiofrequency and cryoablation), ultrasound-guided percutaneous ethanol ablation, transarterial chemoembolization, cementoplasty, and external beam radiotherapy. Thermal ablation has been used to treat metastatic lymph nodes and distant metastasis to the bone, lung, and liver. Radiofrequency thermoablation takes advantage of the heat produced by the radiofrequency generator, while cryoablation alternates cycles of freezing and thawing to destroy tumor cells. These procedures are safe and have a high therapeutic success rate (74, 75). Ultrasound-guided percutaneous ethanol ablation has the main role for neck recurrences (76). Transarterial chemoembolization is used for small and diffuse liver metastases, placing chemotherapy and embolic agents directly into the hepatic artery and permit to treat multiple metastases in the same session treatment, when surgery and local ablative therapy have a limited role (77). In cases of osteolytic bone lesions, cementoplasty has been used to provide bone reinforcement and pain relief (78). In these cases, bisphosphonates (Zoledronic acid) and monoclonal antibodies (Denosumab) may reduce skeletal-related adverse events, such as pathological fractures, metastatic spinal cord compression, and malignant hypercalcemia (79). Finally, external beam radiotherapy was widely used in the past, but its efficacy is questionable, and it may be considered only for palliative purposes in patients with locally advanced disease.

When these local treatments are not feasible or in patients with widespread metastatic disease, a systemic therapy should be initiated. These patients had no effective therapeutic options, until few years ago, but their management has dramatically changed with the availability of tyrosine kinase inhibitors (TKIs). Sorafenib and Lenvatinib, two oral multitargeted TKIs with antiproliferative and antiangiogenetic effects, were approved by the US Food and Drug Administration and European Medicines Agency after the publication of two phase 3, randomized, double-blind, multicenter trials (80, 81). No head-to-head comparison between Sorafenib and Lenvatinib in DTC patients has been conducted so far. In the DECISION trial, Sorafenib significantly prolonged median progression-free survival in patients with advanced DTC (10.8 vs. 5.8 months with placebo) and led to an overall response rate increase (12.2 vs. 0.5%) (80). Similarly, in the SELECT trial, advanced DTC patients treated with Lenvatinib showed a significant prolongation of median progression-free survival in comparison to placebo (18.3 vs. 3.6 months), with a marked improvement in the response rate (64.8 vs. 1.5%). It must be underlined that patients treated with Sorafenib were all TKI naive; by contrast, Lenvatinib represented a second-line TKI therapy for ~25% of treated patients in the trial (81). No significant conclusions can be drawn on overall survival in TKI-treated patients, due to the crossover design of both studies which potentially misrepresents the real effect of the two TKIs. Most of TKI-treated patients experienced adverse events (AEs) in both phase III trials. The most frequent AEs reported on Sorafenib were hand–foot syndrome, diarrhea, alopecia, and rash (80). During treatment with Lenvatinib, more than half of the treated group experienced hypertension, diarrhea, fatigue, and decreased appetite (81). At least for Lenvatinib, AEs seemed to be more severe in older patients (82). In most cases, AE management was achieved by supportive care and by dose reduction or transient drug interruption. Efficacy and tolerability results of the phase III trials were confirmed in the real-life setting (83, 84), leading to an increasing awareness of the management of these patients to prevent major AEs, who are lifelong treated (85, 86). Lenvatinib discontinuation might be necessary in a limited number of cases for toxicity or marked progression. For these patients, salvage therapy with a different TKI is generally recommended, but their availability is restricted to the clinical trial setting or to the “off-label” use (1, 20, 26). Recently, immune checkpoint inhibitors have been tested in preliminary trials for the treatment of advanced thyroid cancer (87). The antitumor activity of the antiprogrammed cell death-1 monoclonal antibody Pembrolizumab has been evaluated in advanced PD-L1-positive DTC with promising results (88).

**Figure 1:**
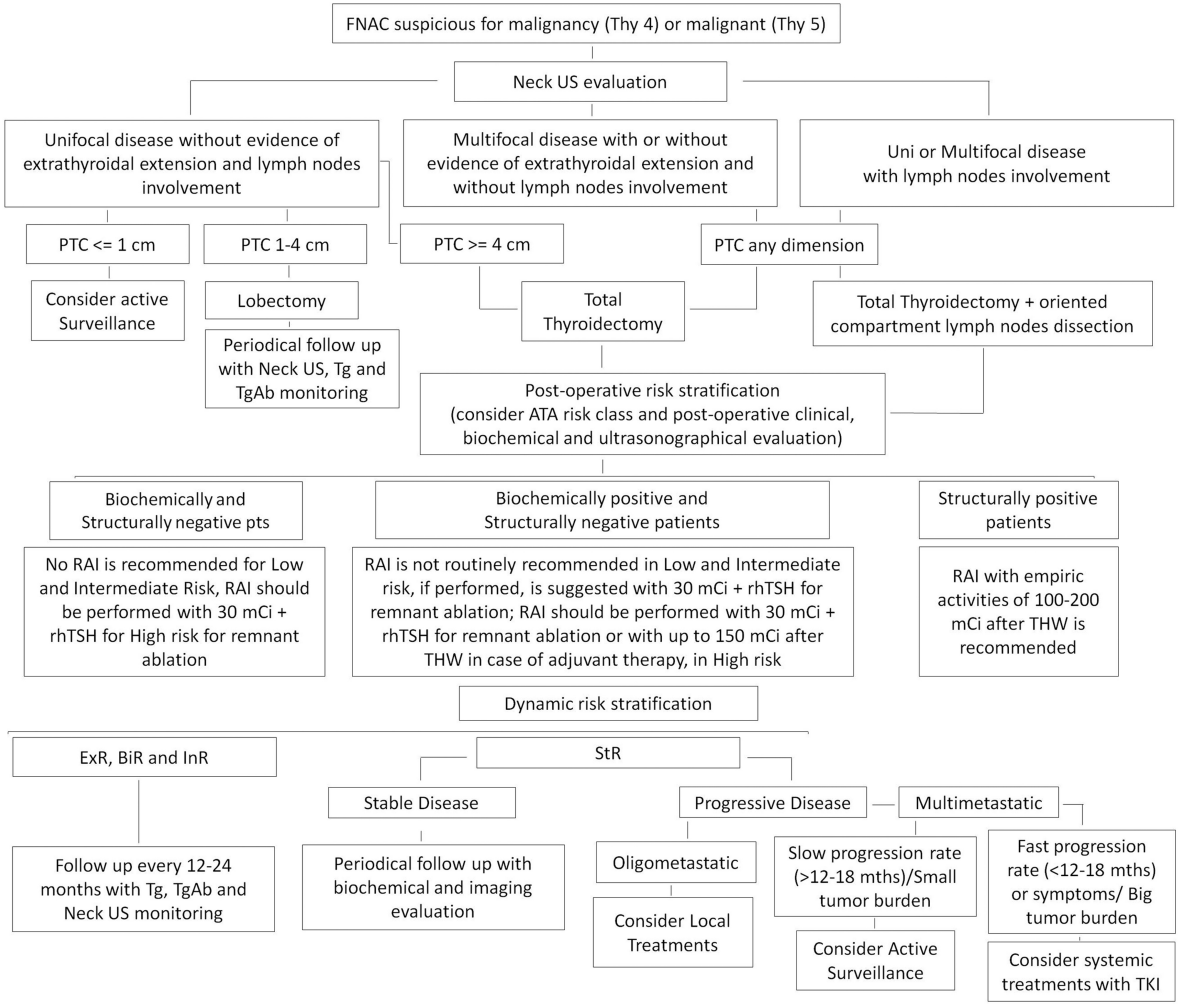
Suggested algorithm for the management of differentiated thyroid cancer (DTC). ExR, Excellent Response; BiR, Biochemical Incomplete Response; InR, Indeterminate Response; StR, Structural Incomplete Response.

A suggested algorithm for the management of DTC from active surveillance to advanced therapies is reported in Figure 1.

## Conclusions

The management of DTC is changing, and currently, a personalized approach, based on an accurate pre- and postoperative risk stratification of the patients, is required. Active surveillance and conservative surgeries are recommended for low risk, while a selective use of radioiodine treatment is advocated for the intermediate-risk patients. The introduction in the clinical practice of TKI treatments allowed to have further options in the treatment of the advanced cases.

## Author Contributions

All authors listed have made a substantial, direct and intellectual contribution to the work, and approved it for publication.

### Conflict of Interest

The authors declare that the research was conducted in the absence of any commercial or financial relationships that could be construed as a potential conflict of interest.
